# Sepsis definitions

**DOI:** 10.5935/0103-507X.20170074

**Published:** 2017

**Authors:** Fernando Suparregui Dias

**Affiliations:** Hospital Pompeia - Caxias do Sul (RS), Brasil.

To the Editor

The recent study in the Revista Brasileira de Terapia Intensiva (RBTI)^(1)^ on the search for consensus on new definitions of sepsis in countries with limited resources represents another attempt to achieve uniformity of the phenotype of a syndrome that is polygenic in nature and thus has a wide variety of presentations. In addition to this biological challenge, we face another barrier when addressing sepsis: interobserver variability. Regardless of the criteria used, the identification of a severely ill patient with a high risk of death, whether due to infection or not, is of irrefutable importance to intensivists, emergency physicians, and all other physicians who provide care to severely ill patients. The same is true for sepsis, and its stratification is essential to identify needs for monitoring, organic support, and infection control. Thus, the ability to identify a patient with sepsis is vital to achieve the best possible outcome. The diagnostic difficulties associated with the sepsis spectrum are not limited to countries with limited resources,^(2)^ as they are also observed in the country with the world's largest economy,^(3)^ even when using antiquated sepsis/septic shock criteria. To illustrate criteria that could improve accuracy in predicting mortality, a recent study of a cohort of patients with sepsis/septic shock from a national reference center^(4)^ revealed an improved performance of the new compared with the previous definitions in terms of predictive accuracy. In this context, improvement of the identification and care of patients with sepsis and septic shock must be addressed. The first measure to address this issue is education and dissemination of knowledge, including in academic institutions, emergency services, inpatient care units, and even intensive care units. A second point to consider is the need for resources to provide care to these patients. It is impossible to improve outcomes, even when using the best health professionals, if we are unable to offer treatment conditions similar to those in developed countries. Finally, health policies, which have been teetering for decades, should seek to establish solutions to reduce sepsis-related mortality in our country. Definitions, criteria, and scores are extremely important. However, given the severity of sepsis, countries with limited resources should not divert their focus from education and the continual search for improving health care as a whole.

*Fernando Suparregui Dias* *Hospital Pompeia - Caxias do Sul (RS), Brazil.*
